# Ethnic differences in post-menopausal plasma oestrogen levels: high oestrone levels in Japanese-American women despiteow weight

**DOI:** 10.1054/bjoc.1999.1082

**Published:** 2000-06

**Authors:** N M Probst-Hensch, M C Pike, R McKean-Cowdin, F Z Stanczyk, L N Kolonel, B E Henderson

**Affiliations:** Department of Preventive Medicine, USC/Norris Comprehensive Cancer Center, University of Southern California, Los Angeles, CA 90033, USA; Department of Obstetrics and Gynecology, University of Southern California, Los Angeles, CA 90033, USA; Institute of Social and Preventive Medicine, University of Basel, Basel, Switzerland; Cancer Research Center, University of Hawaii, Honolulu, HI, 96816, USA

**Keywords:** oestrogen, androstenedione, Japanese-American, breast cancer

## Abstract

Breast cancer incidence in Japanese-American women is approaching that of US Whites. We investigated whether this shift is paralleled by similar post-menopausal plasma hormone levels in the two ethnic groups. We also included African-American and Latina women to further our understanding of possible ethnic differences in oestrogen metabolism. We measured androstenedione (A), oestrone (E1) and oestradiol (E2) in 30 Japanese-American, 39 non-Latina White (‘White’), 66 African-American and 58 Latina women. The (age-adjusted) geometric mean E1 levels were 34 pg ml^−1^in Japanese-Americans, 28 pg ml^−1^in Whites, 35 pg ml^−1^in African-Americans and 31 pg ml^−1^in Latinas. After adjustment for body mass index, Japanese-Americans had the highest mean E1 value of all groups and this was statistically significantly greater than the value for Whites (*P*_t-test_= 0.05). The geometric mean A concentrations were also highest in Japanese-Americans. There was little ethnic difference in E2 levels. In conclusion, post-menopausal plasma oestrogen levels in Japanese-American women are at least as high as those in Whites. © 2000 Cancer Research Campaign


British Journal of Cancer (2000) **82**(11), 1867–1870
© 2000 Cancer Research Campaign
Article no. bjoc.1999.1082, available online at http://www.idealibrary.com on IDEAL®

### Ethnic differences in post-menopausal plasma oestrogen levels: high oestrone levels in Japanese-American women despite low weight

**NM Probst-Hensch[1,3], MC Pike[1], R McKean-Cowdin[1], FZ Stanczyk[2], LN Kolonel[4] and BE Henderson[1]**

[1]Department of Preventive Medicine, USC/Norris Comprehensive Cancer Center, University of Southern California, Los Angeles, CA 90033, USA; [2]Department of Obstetrics and Gynecology, University of Southern California, Los Angeles, CA 90033, USA; [3]Institute of Social and Preventive Medicine, University of Basel, Basel, Switzerland; [4]Cancer Research Center, University of Hawaii, Honolulu, HI 96816, USA

**Summary** Breast cancer incidence in Japanese-American women is approaching that of US Whites. We investigated whether this shift is paralleled by similar post-menopausal plasma hormone levels in the two ethnic groups. We also included African-American and Latina women to further our understanding of possible ethnic differences in oestrogen metabolism. We measured androstenedione (A), oestrone (E1) and oestradiol (E2) in 30 Japanese-American, 39 non-Latina White ('White'), 66 African-American and 58 Latina women. The (age-adjusted) geometric mean E1 levels were 34 pg $ml^{-1}$ in Japanese-Americans, 28 pg $ml^{-1}$ in Whites, 35 pg $ml^{-1}$ in African-Americans and 31 pg $ml^{-1}$ in Latinas. After adjustment for body mass index, Japanese-Americans had the highest mean E1 value of all groups and this was statistically significantly greater than the value for Whites ($P_{\text{t-test}}$ = 0.05). The geometric mean A concentrations were also highest in Japanese-Americans. There was little ethnic difference in E2 levels. In conclusion, post-menopausal plasma oestrogen levels in Japanese-American women are at least as high as those in Whites. © 2000 Cancer Research Campaign

**Keywords:** oestrogen; androstenedione; Japanese-American; breast cancer

Until recently women in Japan were at very low risk of breast cancer (Ursin et al, 1994), particularly in the post-menopausal period when age-specific breast cancer rates remained essentially constant in contrast to the rates in the USA which continue to steadily increase in the post-menopausal period (Pike et al, 1983). Studies of plasma oestrogen concentrations in 'traditional' Asians (living in Asia) compared with Whites have found lower concentrations in the Asians (Goldin et al, 1986; Key et al, 1990; Shimizu et al, 1990). In particular, a study of post-menopausal Japanese women in Miyagi and non-Latina White women in Southern California found a 32% lower level of plasma oestrone (E1) and a 27% lower level of plasma oestradiol (E2) in the Japanese women (Shimizu et al, 1990).

Breast cancer incidence rates in Japanese-American women now approach those of non-Latina White women (Ziegler et al, 1993). We therefore investigated whether post-menopausal plasma oestrogen and androstenedione (A; precursor of E1) levels in Japanese-American women were accordingly similar to those in (non-Latina) White women. The main comparison group was White women, as this group had been used as the comparison group in previous studies. We also included African-American women and Latina women to further our understanding of possible ethnic differences in estrogen metabolism.

*Received 23 June 1999*
*Revised 19 September 1999*
*Accepted 20 September 1999*

*Correspondence to:* NM Probst-Hensch, Institute of Social and Preventive Medicine, Steinengraben 49, CH-4051 Basel, Switzerland

### SUBJECTS AND METHODS

### Subjects

Subjects are part of a multi-ethnic cohort with an emphasis on studying diet and lifestyle characteristics in the aetiology of cancer. Details of this study are described elsewhere (Kolonel et al, 1999). The cohort included 215 251 subjects aged 40–75 years from Hawaii and Los Angeles county. These subjects had responded to a questionnaire mailed between 1993 and 1996. The response rates to the questionnaire were 25.5% in African-Americans, 51.3% in Japanese-Americans, 21.3% in Latinas and 47.0% in Whites. The distributions of educational level and marital status of the cohort broadly resemble those reported by the US Census.

Blood was collected from a random sample of healthy cohort members after receipt of informed consent. Participation in the blood collection phase has been over 70% in all ethnic groups. Eligible controls for this analysis included women who self-reported no history of cancer and in addition must either have experienced a natural menopause, or had both ovaries removed, or had a simple hysterectomy and be at least 55 years of age. Women who reported any hormone use in the 2 weeks before the blood draw were excluded. Sixty-six African-Americans, 30 Japanese-Americans, 58 Latinas and 39 Whites were included in the study. Characteristics of these subjects are described in Table 1.

### Laboratory methods

Plasma aliquots from study subjects were stored in liquid nitrogen until analysis. Plasma levels of A, E1 and E2 were measured by radioimmunoassays previously validated in our laboratory

**Table 1** Characteristics of the study subjects by racial/ethnic group

| Variable | Japanese-Americans | Whites | African-Americans | Latinas |
|---|---|---|---|---|
| No. subjects | 30.0 | 39.0 | 66.0 | 58.0 |
| Mean age, years | 67.2 | 67.9 | 63.6 | 64.8 |
| Mean height, cm | 153.0 | 159.0 | 164.0 | 159.0 |
| Mean weight, kg | 54.6 | 67.3 | 79.0 | 71.2 |
| Mean BMI, kg $m^{-2}$ | 23.3 | 26.7 | 29.4 | 28.2 |
| Nulliparous, % | 6.9 | 5.1 | 4.5 | 7.0 |
| Median age at first birth, years | 26.7 | 26.0 | 22.4 | 24.0 |
| Median age at menarche, years | 13.3 | 13.0 | 13.2 | 13.1 |
| Bilateral oophorectomy, % | 3.3 | 5.1 | 15.2 | 10.4 |

**Table 2** Geometric mean plasma hormone levels by racial/ethnic group[a]

| Hormone | Japanese-Americans | Whites | African-Americans | Latinas |
|---|---|---|---|---|
| Androstenedione (A) | | | | |
| Age-adjusted | 417 | 330 | 380 | 319 |
| | (336–517) | (273–399) | (328–439) | (273–371) |
| Estrone (E1) | | | | |
| Age-adjusted | 34 | 28 | 35 | 31 |
| | (29–39) | (24–32) | (31–39) | (28–35) |
| Age- and BMI-adjusted | 37 | 28 | 34 | 31 |
| | (32–43) | (25–32) | (30–37) | (27–34) |
| Age-, BMI- and A-adjusted | 36 | 28 | 33 | 31 |
| | (31–41) | (25–32) | (30–37) | (28–35) |
| Oestradiol (E2) | | | | |
| Age-adjusted | 15 | 16 | 17 | 16 |
| | (14–17) | (15–17) | (16–18) | (15–17) |
| Age- and BMI-adjusted | 16 | 16 | 16 | 16 |
| | (15–18) | (15–17) | (15–17) | (15–17) |
| Age-, BMI- and A-adjusted | 16 | 16 | 16 | 16 |
| | (15–18) | (15–17) | (15–17) | (15–17) |

[a]All values are given in pg $ml^{-1}$. The associated 95% confidence intervals are given in parentheses.

(Goebelsmann et al, 1973, 1979; Stanczyk et al, 1988; Cassidenti et al, 1992). Prior to quantification, the hormones were first extracted with hexane:ethyl acetate (3:2) and then separated from interfering metabolites by use of Celite column partition chromatography. The assays were each completed in a single run utilizing appropriate quality control samples. The interassay coefficients of variation for A, E1 and E2 were at low levels: 12.2%, 14.5%, 16.3%; at medium levels 8.0%, 8.2%, 8.4%; and at high levels 12.7%, 12.7%, 7.3%.

### Statistical analysis

Statistical analysis was performed on logarithmically transformed values, and geometric mean values are presented. The analysis of covariance (ANCOVA) method was used to assess the impact of ethnicity on hormone concentrations while adjusting for age, body mass index (BMI; weight height$^{-2}$; adjustment for weight or weight/height had similar effects), and, in the case of E1 and E2 analysis, for A. Adjustment for BMI and age were made as continous variables; adjustment for these factors as categorical variables gave similar results. Age was adjusted for as it has previously been reported to be associated with hormone levels (Madigan et al, 1998). Similar results were obtained, though, in the absence of age adjustment. Confounding by oophorectomy, parity, age at first birth, age at menarche, as well as age at and type of menopause, was not observed for any hormone measurement as judged from entering these variables into the ANCOVA models as categorical variables.

Ninety-five per cent confidence intervals, as presented in Table 2, are based on standard errors for ethnic-specific geometric means derived from the ANCOVA models. Two-sided *P*-values presented for the comparison between Japanese-American and White women are based on ANCOVA models only including Japanese-American and White women. Calculations were performed using the PROC GLM procedure for analysis of covariance in the SAS statistical software system (SAS Institute, Cary, NC, USA).

### RESULTS

Age-adjusted geometric mean A levels were highest in Japanese-American women (417 pg $ml^{-1}$), followed by African-Americans, Whites and Latinas (Table 2). These results were not confounded by ethnic differences in BMI or weight. The higher level in Japanese-Americans compared to White women was not statistically significant ($P = 0.08$).

The high Japanese-American plasma A levels were reflected in their high age-adjusted geometric mean plasma E1 level (34 pg $ml^{-1}$), only slightly lower than that of the much heavier African-American women (35 pg $ml^{-1}$), and higher than that of Latina (31 pg $ml^{-1}$) and White women (28 pg $ml^{-1}$). The higher level in Japanese-American compared to White women was not statistically significant ($P = 0.13$). After adjustment for ethnic

 © 2000 Cancer Research Campaign group, the effect of BMI on plasma E1 levels was an increase in $\log_e$ (E1, pg ml$^{-1}$) of 0.023 per 1 kg m$^{-2}$ increase in BMI. After adjustment for BMI, the Japanese-American women had the highest geometric mean E1 levels (37 pg ml$^{-1}$), followed by African-American women, Latinas, and Whites. The higher level in Japanese-American compared to White women became statistically significant after BMI adjustment ($P = 0.05$). The ethnic-specific differences were diminished slightly after adjustment for A levels in addition to BMI.

The age-adjusted plasma E2 concentrations were very similar in the different ethnic groups.

### DISCUSSION

The low oestrogen levels of post-menopausal women living a traditional lifestyle in Japan (Shimizu et al, 1990) were not observed in the Japanese-American women we studied, most of whom (27 of 30) were born in the USA. Geometric mean plasma E1 levels were in fact highest in Japanese-American women, and their geometric mean plasma E2 levels were very close to those of Whites. An increase in blood oestrogen levels in Japanese-American women may provide an explanation for the increase in incidence of breast cancer in Japanese-American women compared to rates in Japan, and, in particular, to the very low rates seen in Japan 30 years ago. The shift in the distribution of menstrual and reproductive factors in Asian-American women towards that in US Whites did not explain much of the increase in breast cancer incidence in Asian-American women (Wu et al, 1996). Causes for the increase in blood estrogen levels in Japanese women living in the USA remain to be elucidated.

Weight and/or obesity are important determinants of plasma oestrogen levels (Potischman et al, 1996; Thomas et al, 1997), as well as of breast cancer risk (Hunter and Willett, 1993; Huang et al, 1997), possibly mediated in part by the increasing extra-glandular aromatization of A to E1 with increasing weight (MacDonald et al, 1978; Simpson et al, 1994). However Japanese-American women in our study, although some 10 kg heavier than 'traditional' post-menopausal women in Japan (Hoel et al, 1983), are still considerably lighter than other American women. In this study they had a 13% lower BMI than Whites. Part of the explanation for their higher E1 levels are the unexpected much higher A levels in Japanese-American women. Genetic variation in the cytochrome P450c17α gene (*CYP17*) has been associated with blood oestrogen levels (Feigelson et al, 1998; Haiman et al, 1999) and may have a role in these high A levels. Ethnic variation in the conversion of A to E1, possibly due to genetic variation in the aromatase gene (Probst-Hensch et al, 1999; Siegelman-Danieli and Buetow, 1999) may further contribute to high E1 levels in Japanese-American women, given that ethnic differences in E1 levels remained after adjustment for A.

Little is known about environmental determinants of endogenous oestrogen and A levels. Some epidemiological evidence found blood oestrogen levels to be positively associated with alcohol intake (Madigan et al, 1998), but Japanese-American women in our cohort consumed much less alcohol than White women (Kolonel et al, 1999). A recent meta-analysis of dietary fat intervention studies found a positive association between dietary fat intake and postmenopausal serum E2 levels (Wu et al, 1999). Yet, Japanese-American women in the multiethnic cohort had the lowest proportion of calories from fat (Kolonel et al, 1999). The effect of soy isoflavones and of soymilk on blood oestrogen levels was investigated in experimental studies (Nagata et al, 1998; Duncan et al, 1999). Results of these studies are only weakly suggestive of a negative association between soy intake and blood oestrogen levels. Soy intake in the Japanese-American women in this study, albeit lower than in Asian women living a traditional lifestyle, was higher than in women of other ethnic backgrounds (Kolonel et al, 1999).

The order of postmenopausal oestrogen and A levels in different ethnic groups did not correlate with the order of increase in the average annual incidence of breast cancer from age 50 to age 70, which was 100% for Latinas, 85% for Whites, 58% for African-Americans and 11% in Japanese-Americans in Los Angeles County between 1988 and 1992 (Parkin et al, 1997). Future studies need to assess more recent changes in age-specific breast cancer rates in different ethnic groups and must investigate the role of genetic and environmental factors in determining ethnic differences in blood oestrogen and A levels. We will be able to address a number of these issues as data accumulate in the Multiethnic Cohort.

### ACKNOWLEDGEMENTS

This research was supported by funds from the California Breast Cancer Research Program of the University of California (Grant 21B-0019), from the Public Health Service (National Cancer Institute) (Grant R01 CA 54281), and from the Swiss National Science Foundation (3200–55 142.98).

### REFERENCES

Cassidenti DL, Pike MC, Vijod AG, Stanczyk FZ and Lobo RA (1992) A reevaluation of estrogen status in postmenopausal women who smoke. *Am J Obstet Gynecol* **166**: 1444–1448

Duncan AM, Merz BE, Xu X, Nagel TC, Phipps WR and Kurzer MS (1999) Soy isoflavones exert modest hormonal effects in premenopausal women. *J Clin Endocrinol Metab* **84**: 192–197

Feigelson HS, Shames LS, Pike MC, Coetzee GA, Stanczyk FZ and Henderson BE (1998) Cytochrome P450c17α gene (*CYP17*) polymorphism is associated with serum estrogen and progesterone concentrations. *Cancer Res* **58**: 585–587

Goebelsmann U, Horton R, Mestman JH, Arce JJ, Nagata Y and Nakamura RM (1973) Male pseudohermaphroditism due to testicular 17β-hydroxysteroid dehydrogenase deficiency. *J Clin Endocrinol Metab* **36**: 867–879

Goebelsmann U, Bernstein GS, Gale JA, Kletzky OA, Nakamura RM and Coulson AH (1979) Serum gonadotropin, testosterone, estradiol and estrone levels prior to and following bilateral vasectomy. In: *Vasectomy: Immunologic and Pathophysiologic Effects in Animals and Man*, Lepow JH and Crozier R (eds), pp. 165–175. Academic Press: New York

Goldin BR, Adlercreutz H, Gorbach SL, Woods MN, Dwyer JT, Conlon T, Bohn E and Gershoff SN (1986) The relationship between estrogen levels and diets of Caucasian American and Oriental immigrant women. *Am J Clin Nutr* **44**: 945–953

Haiman CA, Hankinson SE, Spiegelman D, Colditz GA, Willett WA, Speizer FE, Kelsey KT and Hunter DJ (1999) The relationship between a polymorphism in CYP17 with plasma hormone levels and breast cancer. *Cancer Res* **59**: 1015–1020

Hoel DG, Wakabayashi T and Pike MC (1983) Secular trends in the distributions of the breast cancer risk factors: menarche, first birth, menopause and weight, in Hiroshima and Nagasaki, Japan. *Am J Epidemiol* **118**: 78–79

Huang Z, Hankinson SE, Colditz GA, Stampfer MJ, Hunter DJ, Manson JE, Hennekens CH, Rosner B, Speizer FE and Willett WC (1997) Dual effects of weight and weight gain on breast cancer risk. *JAMA* **278**: 1407–1411

Hunter DJ and Willett WC (1993) Diet, body size, and breast cancer. *Epidemiol Rev* **15**: 110–132

Key TJA, Chen J, Wang DY, Pike MC and Boreham J (1990) Sex hormones in women in rural China and in Britain. *Br J Cancer* **62**: 631–636

*© 2000 Cancer Research Campaign* 

Kolonel LN, Henderson BE, Hankin JH, Nomura AMY, Wilkens LR, Pike MC, Stram DO, Monroe KR, Earle ME and Nagamine FS (1999) A multiethnic cohort in Hawaii and Los Angeles: baseline characteristics. *Am J Epidemiol* (in press)

MacDonald PC, Edman CD, Hemsell DL, Porter JC and Siiteri PK (1978) Effect of obesity on conversion of plasma androstenedione to estrone in postmenopausal women with and without endometrial cancer. *Am J Obstet Gynecol* **130**: 448–454

Madigan MP, Troisi R, Potischman N, Dorgan JF, Brinton LA and Hoover RN (1998) Serum hormone levels in relation to reproductive and lifestyle factors in postmenopausal women (United States). *Cancer Causes Control* **9**: 199–207

Nagata C, Takatsuka N, Inaba S, Kawakami N and Shimizu H (1998) Effect of soymilk consumption on serum estrogen concentrations in premenopausal Japanese women. *J Natl Cancer Inst* **90**: 1830–1835

Parkin DM, Whelan SL, Ferlay J, Raymond L and Young J (eds) (1997) *Cancer Incidence in Five Continents, Volume VII*. IARC Scientific Publications No. 143. IARC: Lyon

Pike MC, Krailo MD, Henderson BE, Casagrande JT and Hoel DG (1983) 'Hormonal' risk factors, 'breast tissue age' and the age-incidence of breast cancer. *Nature* **303**: 767–770

Potischman N, Swanson CA, Siiteri P and Hoover RN (1996) Reversal of relation between body mass and endogenous estrogen concentrations with menopausal status. *J Natl Cancer Inst* **88**: 756–758

Probst-Hensch NM, Ingles SA, Diep AT, Haile RW, Stanczyk FZ, Kolonel LN and Henderson BE (1999) Aromatase and breast cancer susceptibility. *Endocrine-Related Cancer* **6**: 165–173

Shimizu H, Ross RK, Bernstein L, Pike MC and Henderson BE (1990) Serum oestrogen levels in postmenopausal women: comparison of American whites and Japanese in Japan. *Br J Cancer* **62**: 451–453

Siegelmann-Danieli N and Buetow KH (1999) Constitutional genetic variation at the human aromatase gene (*Cyp19*) and breast cancer risk. *Br J Cancer* **79**: 456–463

Simpson ER, Mahendroo MS, Means GD, Kilgore MW, Hinshelwood MM, Graham-Lorence S, Amerneh B, Ito Y, Fisher CR, Michael MD, Mendelson CR and Bulun SE 1994) Aromatase cytochrome P450, the enzyme responsible for estrogen iosynthesis. *Endocrine Rev* **15**: 342–355

Stanczyk FZ, Shoupe D, Nunez V, Macias-Gonzales P, Vijod MA and Lobo RA (1988) A randomized comparison of nonoral estradiol delivery in postmenopausal women. *Am J Obstet Gynecol* **159**: 1540–1546

Thomas HV, Key TJ, Allen DS, Moore JW, Dowsett M, Fentiman IA and Wang DY (1997) Re: Reversal of relation between body mass and endogenous estrogen concentrations with menopausal status. *J Natl Cancer Inst* **89**: 396–397

Ursin G, Bernstein L and Pike MC (1994) Breast cancer. In: *Trends in Cancer Incidence and Mortality*, Doll R, Fraumeni J and Muir CS (eds), *Cancer Surv* 19/20

Wu AH, Ziegler RG, Pike MC, Nomura AMY, West DW, Kolonel LN, Horn-Ross PL, Rosenthal JF and Hoover RN (1996) Menstrual and reproductive factors and risk of breast cancer in Asian-Americans. *Br J Cancer* **73**: 680–686

Wu AH, Pike MC and Stram O (1999) Meta-analysis: dietary fat intake, serum estrogen levels, and the risk of breast cancer. *J Natl Cancer Inst* **17**: 529–534

Ziegler RG, Hoover RN, Pike MC, Hildesheim A, Nomura AMY, West DW, Wu-Williams AH, Kolonel LN, Horn-Ross PL, Rosenthal JF and Hyer MB (1993) Migration patterns and breast cancer risk. *J Natl Cancer Inst* **85**: 1819–1827

 *© 2000 Cancer Research Campaign*
